# Next-Generation “-omics” Approaches Reveal a Massive Alteration of Host RNA Metabolism during Bacteriophage Infection of *Pseudomonas aeruginosa*

**DOI:** 10.1371/journal.pgen.1006134

**Published:** 2016-07-05

**Authors:** Anne Chevallereau, Bob G. Blasdel, Jeroen De Smet, Marc Monot, Michael Zimmermann, Maria Kogadeeva, Uwe Sauer, Peter Jorth, Marvin Whiteley, Laurent Debarbieux, Rob Lavigne

**Affiliations:** 1 Institut Pasteur, Molecular Biology of the Gene in Extremophiles Unit, Department of Microbiology, Paris, France; 2 Université Paris Diderot, Sorbonne Paris Cité, Cellule Pasteur, Paris, France; 3 Laboratory of Gene Technology, Department of Biosystems, KU Leuven, Leuven, Belgium; 4 Institut Pasteur, Laboratoire Pathogenèse des bactéries anaérobies, Département de Microbiologie, Paris, France; 5 Institute of Molecular Systems Biology, Eidgenössische Technische Hochschule (ETH) Zürich, Zürich, Switzerland; 6 Department of Molecular Biosciences, Institute for Cellular and Molecular Biology, Center for Infectious Disease, University of Texas, Austin, Texas, United States of America; Aix-Marseille Université, Centre National de la Recherche Scientifique, FRANCE

## Abstract

As interest in the therapeutic and biotechnological potentials of bacteriophages has grown, so has value in understanding their basic biology. However, detailed knowledge of infection cycles has been limited to a small number of model bacteriophages, mostly infecting *Escherichia coli*. We present here the first analysis coupling data obtained from global next-generation approaches, RNA-Sequencing and metabolomics, to characterize interactions between the virulent bacteriophage PAK_P3 and its host *Pseudomonas aeruginosa*. We detected a dramatic global depletion of bacterial transcripts coupled with their replacement by viral RNAs over the course of infection, eventually leading to drastic changes in pyrimidine metabolism. This process relies on host machinery hijacking as suggested by the strong up-regulation of one bacterial operon involved in RNA processing. Moreover, we found that RNA-based regulation plays a central role in PAK_P3 lifecycle as antisense transcripts are produced mainly during the early stage of infection and viral small non coding RNAs are massively expressed at the end of infection. This work highlights the prominent role of RNA metabolism in the infection strategy of a bacteriophage belonging to a new characterized sub-family of viruses with promising therapeutic potential.

## Author Summary

The increase of the proportion of multidrug resistant bacterial strains is alarming and alternative ways to treat infections are necessary such as the use of the natural enemies of bacteria, also known as phage therapy. However, explorations of the molecular mechanisms underlying the viral cycle of bacteriophages have been so far restricted to a small number of viruses infecting model bacteria such as *Escherichia coli*. By combining next-generation transcriptomics and metabolomics approaches, we have now demonstrated that the virulent bacteriophage PAK_P3, infecting the opportunistic pathogen *Pseudomonas aeruginosa*, directly interferes with specific host metabolic pathways to complete its infection cycle. In particular, it triggers a dramatic degradation of host RNAs and stimulates bacterial pyrimidine metabolism to promote a nucleotide turnover. Overall, we found that upon PAK_P3 infection, host metabolism is redirected to generate the required building blocks for efficient viral replication. We also showed that PAK_P3 gene expression relies on RNA-based regulation strategies using small non coding RNAs and antisense RNAs. Our findings highlight the molecular strategies employed by this virulent phage, which is a representative of a new subfamily of viruses shown to display promising therapeutic values.

## Introduction

The threat of antibiotic resistance has renewed attention to phage therapy leading to isolation of many bacteriophages (phages) targeting human pathogens such as *Pseudomonas aeruginosa* and, consequently, an increasing number of phage genome sequences are available [1]. Comparative genomics has allowed the implementation of a genome-based taxonomy for tailed phages which reflects their great diversity. However, the lack of knowledge of molecular mechanisms underlying their infectious cycles is slowing down their global acceptance as valid therapeutics. Indeed, outside basic characterizations (e.g. phage growth parameters, identification of bacterial receptors and phage structural proteins) many questions about their infection strategy remain conspicuously unanswered for most phages, mainly because genome annotations cannot provide hints on the functions of many viral genes.

For *Pseudomonas* phages, the introduction of whole-transcriptome studies with RNA-Sequencing (RNA-Seq) has recently led to improved genome annotations, discovery of regulatory elements and elucidation of temporal transcriptional schemes, while at the same time looking at the impact on transcription regulation of host genes upon phage infection. For example, giant phage ϕKZ is now understood to infect and lyse its host cell as well as produce phage progeny in the absence of functional bacterial transcriptional machinery [2]. The impact of phage infection on the host can also be observed at the metabolome level. Recently, a high coverage metabolomics analysis comparing several viruses that cover most genera of *Pseudomonas* phages infecting strain PAO1, revealed specific phage and infection-stage alterations of the host physiology. These changes often appear mediated by phage-encoded auxiliary metabolic genes (AMGs) and by host gene features that are specifically modulated by the phage [3].

One *Pseudomonas* phage clade that has not yet been studied is comprised of the two newly proposed genera (*PAK_P1-like* and *KPP10-like*) belonging to a new subfamily of viruses, *Felixounavirinae*. Interestingly, these phages display the best therapeutic potential in an experimental murine lung infection model as compared to other *P*. *aeruginosa* phages belonging to distinct clades [4]. Aside from structural genes, most of their predicted ORFs could not be associated with a putative function and consequently, no meaningful conclusions about their strategy for hijacking host metabolism could be drawn [5].

In this work, we used synergistic next generation approaches to provide the first parallel transcriptomics and metabolomics analyses on phage PAK_P3, a representative of the *KPP10-like* genus. We intended to draw a detailed global scheme of PAK_P3 infectious cycle by addressing the following questions: Does PAK_P3 control expression of specific bacterial genes? Does it interfere with bacterial metabolism? How does it regulate its gene expression?

Our major finding is the predominant role of RNA metabolism in PAK_P3 infectious strategy. Beside the dramatic global depletion of host transcripts induced by phage infection, PAK_P3 causes a strong up-regulation of a single specific host operon. Consistently, an increase of pyrimidine metabolism upon infection was revealed by metabolomics analysis showing that, like T-even phages, PAK_P3 actively manages nucleotides scavenged from their hosts [6]. In addition, besides revealing the temporal expression of PAK_P3 genes, we highlighted an unexpected prominent role of RNA-based regulation of phage gene expression. Indeed, PAK_P3 produces early antisense transcripts encompassing structural genes as well as phage-encoded small non coding RNAs.

## Results

### Reannotation of strain PAK genome using transcriptomic data revealed numerous RNA-based regulatory elements

To study bacterial transcriptional response to PAK_P3 infection, it was first crucial to exhaustively characterize the genome of its host, *P*. *aeruginosa* strain PAK. Initially, a draft genome was produced and assembled (6.28 Mbp, 66.3% GC content and 6,267 predicted ORFs). Next, a detailed genome reannotation was performed based on RNA-Seq data generated from exponentially growing and uninfected PAK cells (S1 Table).

Using COV2HTML [7] to visualize transcripts, we manually reannotated 32 open reading frames (ORFs) (by detection of an alternative start codon) and defined 63 new putative coding sequences. Among them, 39 have been previously annotated in other *P*. *aeruginosa* genomes while the other 24 are new hypothetical coding sequences that display no homology to sequences in databases and may be considered as strain-specific (S1 Table).

Recent genome-wide studies based on RNA-Seq led to the discovery of a substantial number of non-coding RNAs (ncRNAs), which are now acknowledged as important modulators of various bacterial processes (for a review of ncRNAs in *Pseudomonas aeruginosa*, see [8]). We identified a total of 75 small ncRNAs encoded in the PAK genome, 26 of which correspond to known functional classes (S1 Fig, S1 Table). Among these 26, 12 are similar to uncharacterized ncRNA conserved within the *Pseudomonas* genus and the other 14 have predicted functional assignments, according to Rfam. The majority of ncRNAs (49 out of 75) could not be assigned to any functional class (see Methods), and have not been identified in previous RNA-Seq investigations carried out on *P*. *aeruginosa* strains PAO1 and PA14 [9–11], suggesting that they may represent novel ncRNAs regulators.

Eighteen long antisense RNAs (asRNAs) were also identified within genes. As they do not display any consistent ORFs, they are not likely to contain overlapping protein-coding genes and may therefore *cis*-interfere with the expression of gene they are encoded in (S1 Fig, S1 Table). Finally, 32 potential riboswitches were identified by looking at intergenic transcription events starting at a significant distance from a downstream gene, usually involved in a metabolic pathway and displaying a characteristic RNA-Seq pattern. Eleven of them were confirmed by Rfam search (S1 Fig, S1 Table).

With more than 50% of new ncRNAs amongst total ncRNAs identified, along with the identification of new putative riboswitches and evidence of antisense transcripts, strain PAK exemplifies the great diversity of bacterial RNA-based regulation [12]. Such in-depth annotation, including new strain-specific RNA elements, was mandatory for the subsequent transcriptomic analysis of phage infected cells in order to assess the impact of phage infection on host physiology.

### The fast-replicating phage PAK_P3 progressively takes over host cell transcription and tends to dominate over competing mobile genetics elements

**Fig 1:**
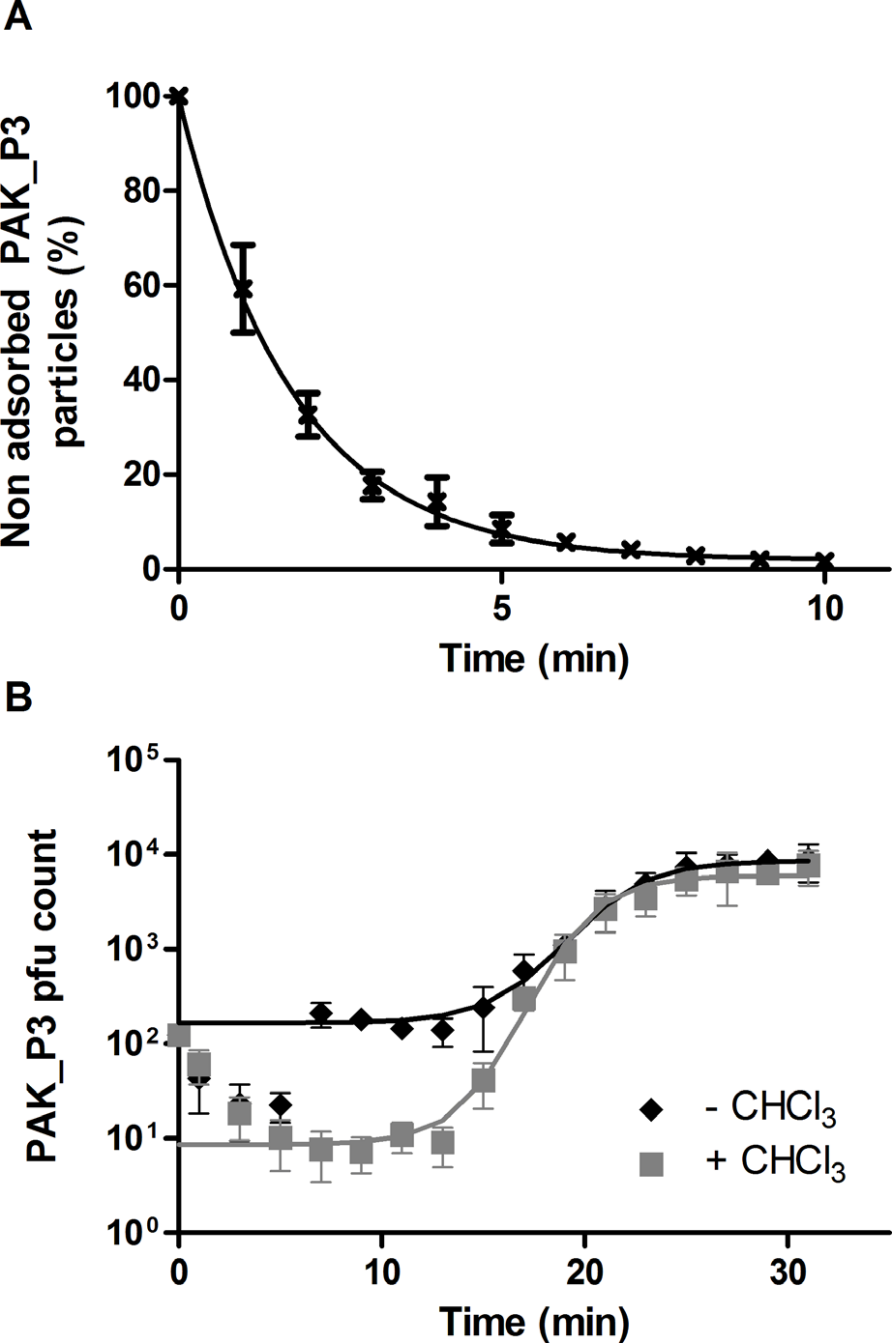
PAK_P3 rapidly adsorbs to its host and efficiently produces new progenies. **(A)** Adsorption assays of PAK_P3 on *P*. *aeruginosa* strain PAK. **(B)** One-step growth curve of PAK_P3. Samples treated with (grey squares) or without (black diamonds) CHCl_3_. A logistic regression was used to fit the data. Four independent experiments were combined and data are presented as means with standard deviations.

To study the dynamics of the transcriptional and metabolic consequences of phage infection, we first selected the most relevant time points, representative of the different steps of the course of infection by determining the growth parameters of PAK_P3. Adsorption assays revealed that ≥90% of PAK_P3 virions adsorbed on strain PAK within 4.6 ±0.7 min (k_a_ = 2.2.10^- 9^ ±5.1.10^−10^ mL.min^-1^) (Fig 1A). A standard one step growth experiment showed that the first functional new virions are rapidly assembled (eclipse period: 12.3 ±0.4 min) and almost immediately released (latency period: 13 ±2.1 min), producing an average of 53 ±21 progeny phages per infected cell (Fig 1B). With a mean infection cycle duration as short as 18 ±0.6 min, PAK_P3, with a genome length ≥80 kb, is faster than the myoviruses ϕKZ (60–65 min, 280 kb) [3] and T4 (25–30 min, 168 kb) [13], therefore being among the most rapid *Myoviridae*.

**Fig 2:**
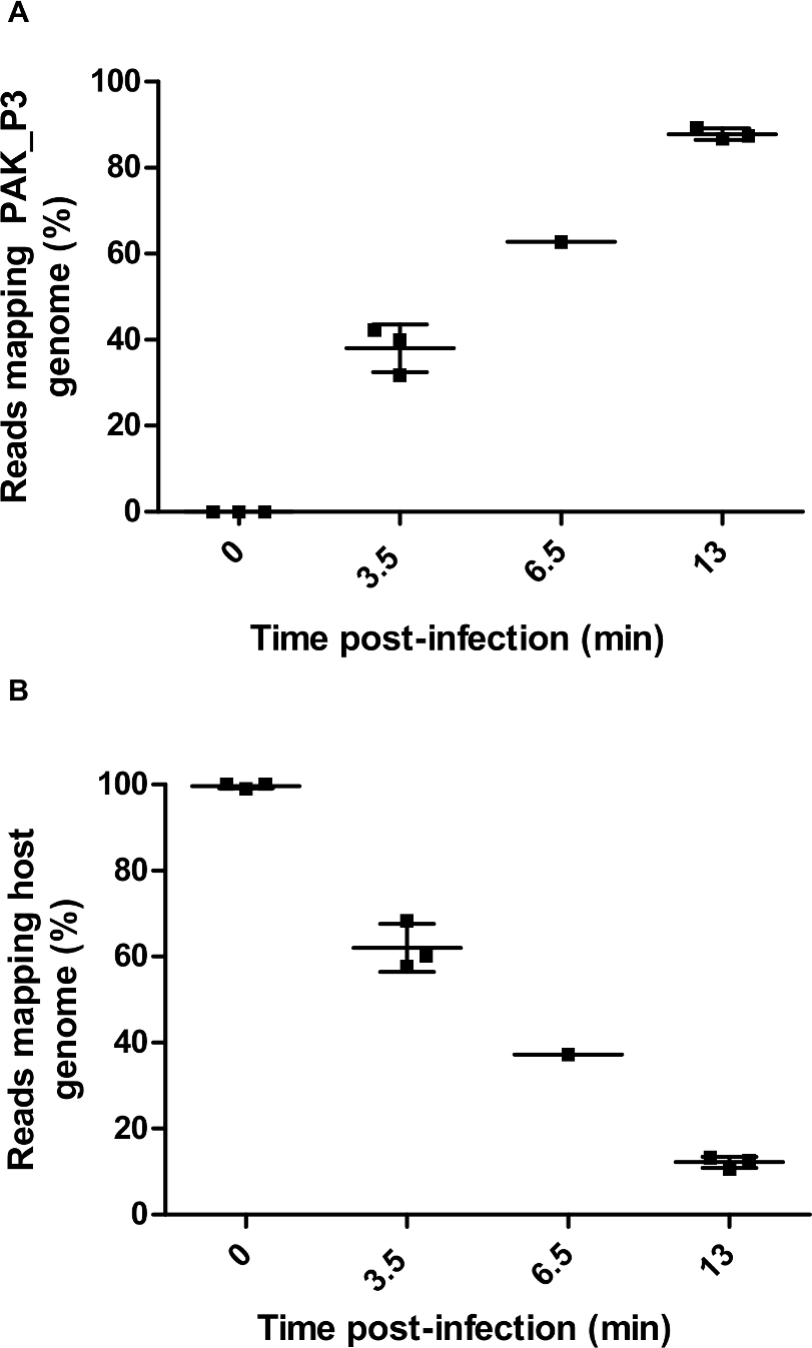
PAK_P3 takes over the host cell transcription over the course of infection. Three independent biological replicates of RNA extracts were harvested from PAK_P3 infected cells at 0min, 3.5min and 13min post infection, as well as a single sample collected at 6.5min and all were subsequently sequenced. Plotting the percentage of reads mapping to the PAK_P3 genome **(A)** and to the host genome **(B)** over the course of infection shows that PAK_P3 progressively dominates the transcriptional environment of the cell with phage transcripts.

Given the short eclipse period duration, we focused on 3.5 min and 13 min time points as representative snapshots of the beginning (early) and the end (late) of one infection cycle at the transcription level. Investigation of the regulation of both viral and host gene expression over a single phage infection cycle by RNA-Seq revealed a progressive and dramatic replacement of host mRNA with phage transcripts. This process eventually results in host transcripts representing fewer than 13% of non-ribosomal RNAs in the cell (Fig 2). However, even in the context of this dramatic depletion of host transcripts, a response to phage infection at the transcription level was observed, suggesting a globally accelerated degradation of unstable mRNA species rather than a global transcriptional repression as described for phage T4 [14].

**Fig 3:**
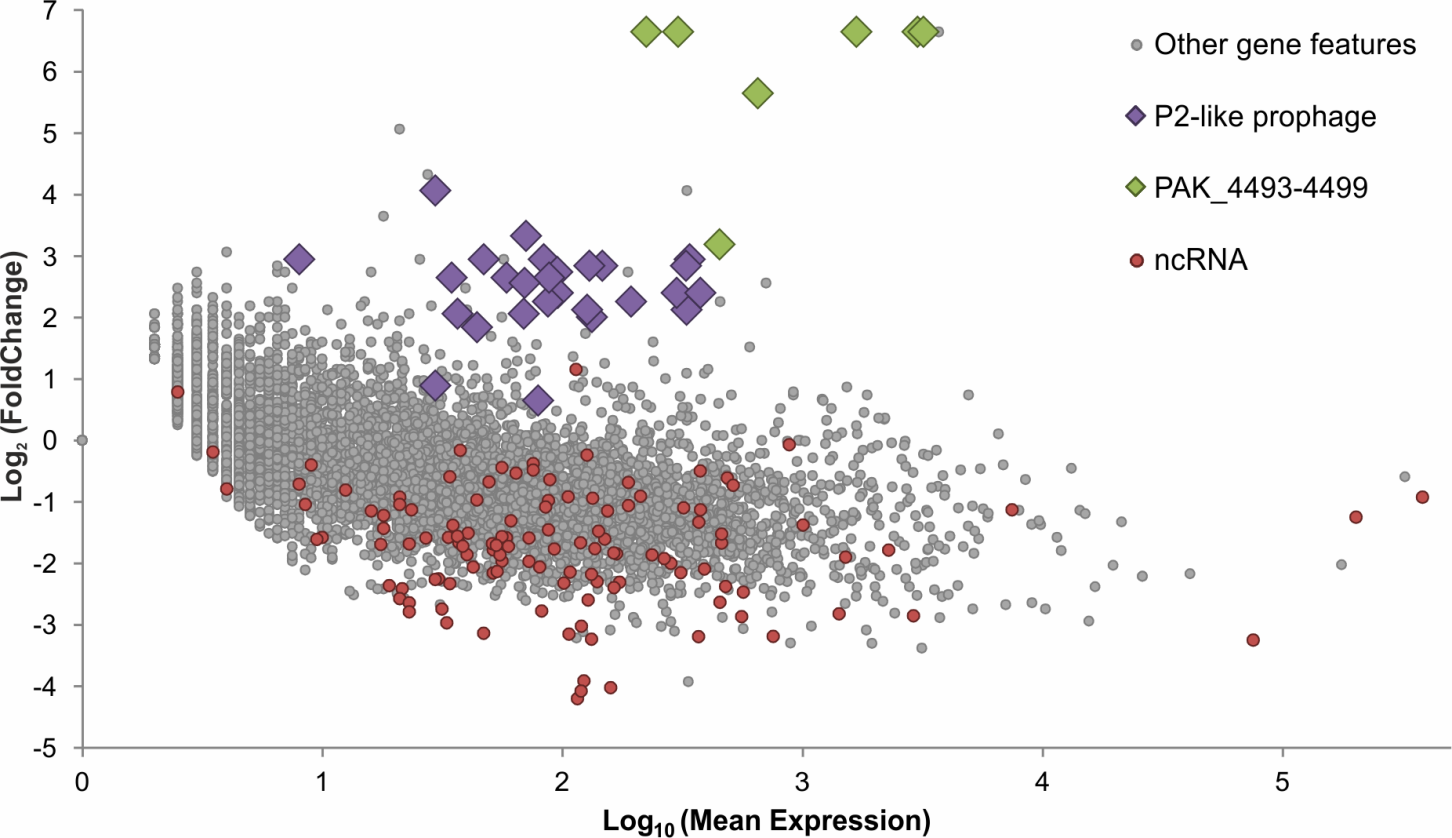
PAK_P3 alters expression of many host gene features by late infection. Differential expression analysis of host gene features comparing transcript abundance between phage negative controls (t = 0 min) and late infection (t = 13min) was performed. This comparison was made after normalizing the read counts that map to each host gene feature between both conditions, ignoring reads that map to the phage, which artificially enriches reads in the late condition. This method thus compares the negative control directly to a host transcript population that has been depleted by replacement with phage transcripts during infection, normalizing away the global depletion so that more specific shifts can be reported and independently tested for.

In addition to providing a transcriptional environment fully co-opted by the phage for optimal infection (*i*.*e*. making host RNA polymerase available for viral RNAs for instance), this observed host RNA depletion can be expected to suppress host defenses that require host transcripts to function [15] as well as prophage induction attempts. Indeed, PAK_P3 infection appears to activate the transcription of a P2-like prophage (Fig 3, S2 Table) as corresponding transcripts display a 6.8-fold increase in PAK_P3 infected cells at late time point compared to non-infected cells. However, host transcripts overall were depleted by 7.2 fold at the late time point, which would leave the infected cell with marginally fewer prophage transcripts than during exponential growth, indicating that the transcriptional activation of the prophage is suppressed, although not completely blocked.

### Phage infection triggers dramatic and specific up-regulation of one bacterial operon linked to RNA processing

Although host transcripts are globally replaced by phage transcripts, we could still analyze the changes in host mRNA population by normalizing the host transcript counts before infection to the counts after infection, artificially depleting counts before infection and enriching reads after infection. This allows us to look for specific differential expression of host gene features in response to the stress of phage infection as well as specific changes in host gene expression imposed by the phage in order to hijack cellular metabolism.

We discovered that one operon, comprising six genes (PAK_4493–4499), has a nearly 80-fold increase in abundance relative to other host genes, which is large enough to strongly enrich its transcript abundance relative to the total RNA in the cell even in the context of global RNA degradation (Fig 3, S2 Table).

RNA-Seq analysis thus provided precise depictions of phage influence on the bacterial transcriptome and host transcriptional response to infection. It also allowed us to decipher the transcriptional strategy adopted by the phage to control its own gene expression (see below). To have a broader view of the consequences of a phage infection on host cell physiology, we performed a complementary metabolomics analysis.

### Amino acid, nucleotide/sugar and pyrimidine pathways display drastic changes in phage infected cells

Viruses depend on host cell metabolic resources to complete their intracellular parasitic development [16]. However, the effects of phage infection on host metabolism are still poorly understood. We thus investigated whether the phage completely shuts off host metabolism, as it may burden efficient phage replication, or if it influences specific pathways.

**Fig 4:**
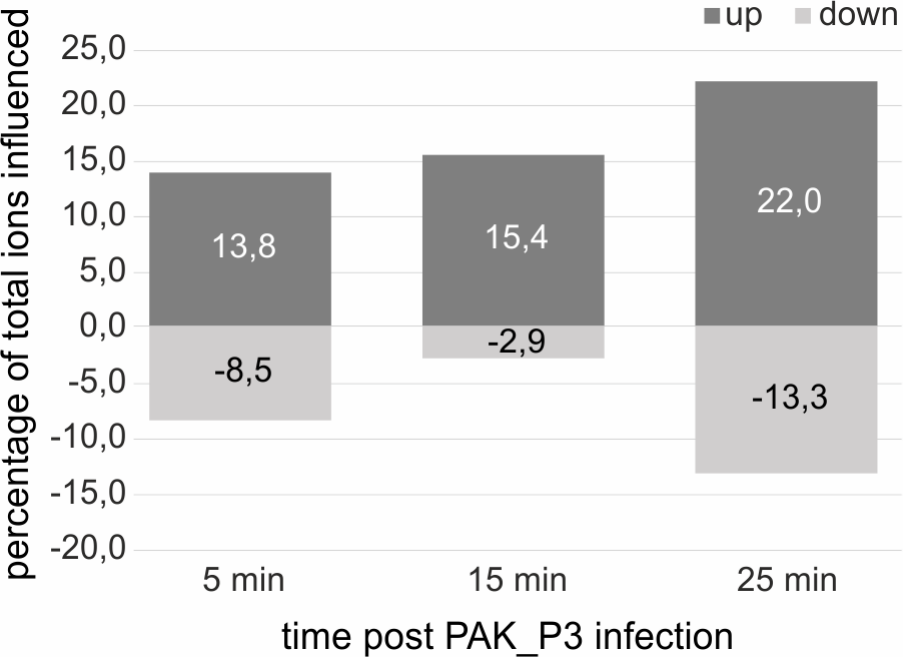
PAK_P3 alters *P*. *aeruginosa* metabolite content over the course of infection. Percentage of altered *Pseudomonas* metabolite ions during the course of infection (p-value ≤ 0,05, │Log_2_(fold change)│ ≥ 0,5), y-axis shows percentage and x-axis shows the time points during infection. In total 377 ions were measured.

To assess the impact of PAK_P3 infection on strain PAK metabolism, high-coverage metabolomics analysis was applied to monitor metabolite dynamics during infection [17]. Comparison of the metabolite levels at different time points post infection to uninfected samples revealed significant metabolic changes upon phage infection. Within the first 5 min of infection, 22% of measured metabolites display altered levels with 13.8% increased and 8.5% decreased (p-value ≤ 0,05, │Log_2_(fold change)│ ≥ 0,5). The proportion of metabolites with increased levels gradually rises up to 22% at 25 min post infection, while the proportion of metabolites with decreased levels temporarily drops to 3% to finally increase back to 13% during bacterial lysis (Fig 4). These variations indicate that PAK_P3 does not simply deplete available host metabolites but relies on an active metabolism in agreement with recent observations identifying phage-specific physiological alterations [3,18].

**Fig 5:**
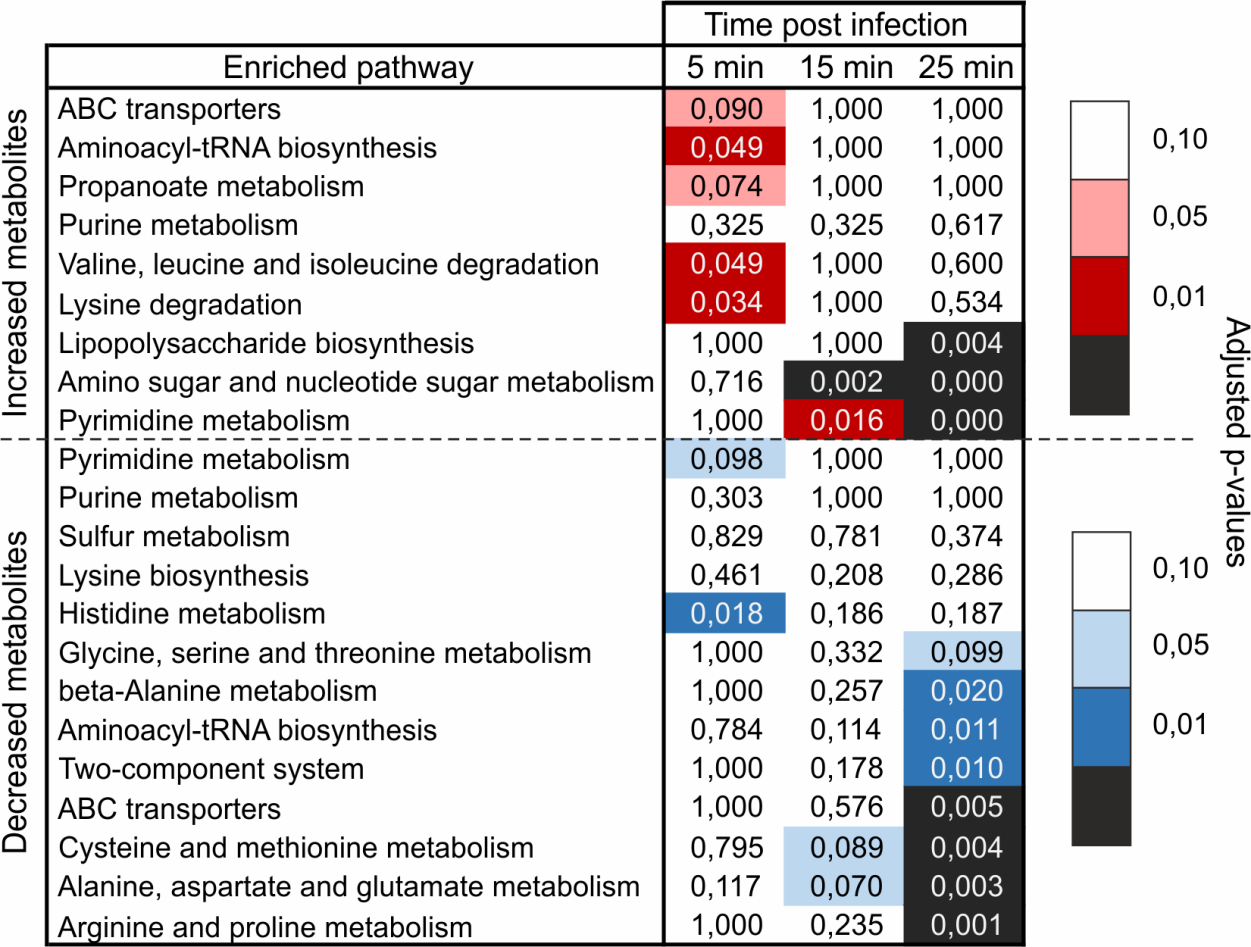
Pathway enrichment analysis during PAK_P3 infection revealed its requirement on pyrimidine metabolism. Values of the enrichment analysis are shown for the significantly enriched pathways (rows) at the different time points (columns). The color scale indicates the cut-off for the p-values, where red and blue are used for, respectively, the pathways found enriched among increased and decreased metabolites.

Next, to investigate whether PAK_P3 targets specific metabolic pathways, a metabolite set enrichment analysis was performed. Overall, metabolites from amino/nucleotide sugar and pyrimidine metabolic pathways were found over-represented among increasing metabolites, while amino acid-related pathways were enriched among decreasing metabolites at later stages of infection (Fig 5). Intriguingly, about 50% of the detected (deoxy)nucleotides-phosphates have at least two-fold increased levels during infection (S3 Table).

Among accumulating metabolites belonging to amino/nucleotide sugar metabolism and to lipopolysaccharide biosynthesis pathway (Fig 5), it is worth noting that the levels of cell wall precursors such as UDP-N-acetyl-D-glucosamine or UDP-N-acetyl-D-galactosaminuronic acid show a significant two- and three-fold increase, respectively, during late infection (S4 Table). This increase is not accompanied by altered expression of host genes involved in this pathway (see below).

Most enriched pathways among decreasing metabolites involve amino acid biosynthesis (Fig 5), more specifically Arg, Pro, Ala, Asn, Glu, Cys and Met metabolism were found significantly enriched (p-value < 0.005). These observed decreases may indicate drainage of amino acid pools in the cell during phage particle formation, due to an imbalance between cellular amino acid biosynthesis and consumption by the phage.

### Phage-induced changes of host metabolome composition are not otherwise mediated through differential expression of host genes

**Fig 6:**
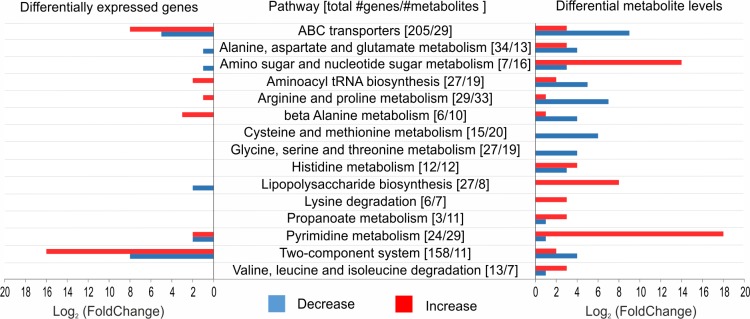
Comparison of significant changes in transcriptomics and metabolomics data from PAK_P3 infected cells reveals no direct correlation. On the left, the number of genes with a significant differential expression (│Log_2_(fold change)│ > 1.3, p-value < 0.05) is shown. On the right, an overview of the number of metabolites with significantly changed levels (│Log_2_(fold change)│ > 0.5, p-value < 0.05) is shown. The middle column entails all studied metabolic pathways and indicated between brackets are, respectively, the total number of genes and metabolites involved in this pathway. Red = increase in transcript/metabolite level, Blue = a decrease in transcript/metabolite level.

We initially hypothesized that the observed changes in metabolome composition upon infection would largely be the result of a differential expression of host genes induced by the phage. This would indicate that PAK_P3 mainly interferes with cellular transcription to alter host physiological processes. To address this question, we investigated if the variations at the metabolome level could be directly linked to transcriptional changes. We thus analyzed all metabolites belonging to pathways highlighted by the pathway enrichment analysis (see above) that display significant variations (│Log_2_(fold change)│ > 0.5, p-value < 0.05) as well as differential expression of coding sequences related to the corresponding pathways with a stringent cut-off point (│Log_2_(fold change)│ > 1.3, p-value < 0.05) (Fig 6).

Only few genes linked to these pathways were significantly differentially expressed upon late infection, indicating that the phage influence on host metabolism is not primarily mediated through differential gene expression. In fact, several pathways with increased metabolite levels have a decreased transcription of the involved genes or vice versa (e.g. lipopolysaccharide biosynthesis).

Based on these complementary “-omics” approaches, it can be concluded that PAK_P3 does not otherwise redirect host physiology towards viral reproduction through modification of host gene expression. The general degradation of host RNA observed likely ensures sufficient building blocks for viral genome replication. The metabolic content of PAK_P3 infected cells shows both increased and decreased metabolite levels. We hypothesize these changes are either the direct consequence of an increased viral consumption of metabolites (e.g. amino acid metabolism) or are likely triggered by phage-encoded AMGs (e.g. pyrimidine metabolism).

### Gene expression of PAK_P3 is temporally regulated

Besides redirecting host cell physiology, the phage must also control its own gene expression. Here we intended to investigate the transcriptional strategy of PAK_P3 and also discovered unexpected regulatory mechanisms the phage uses to complete its infection cycle.

**Fig 7:**
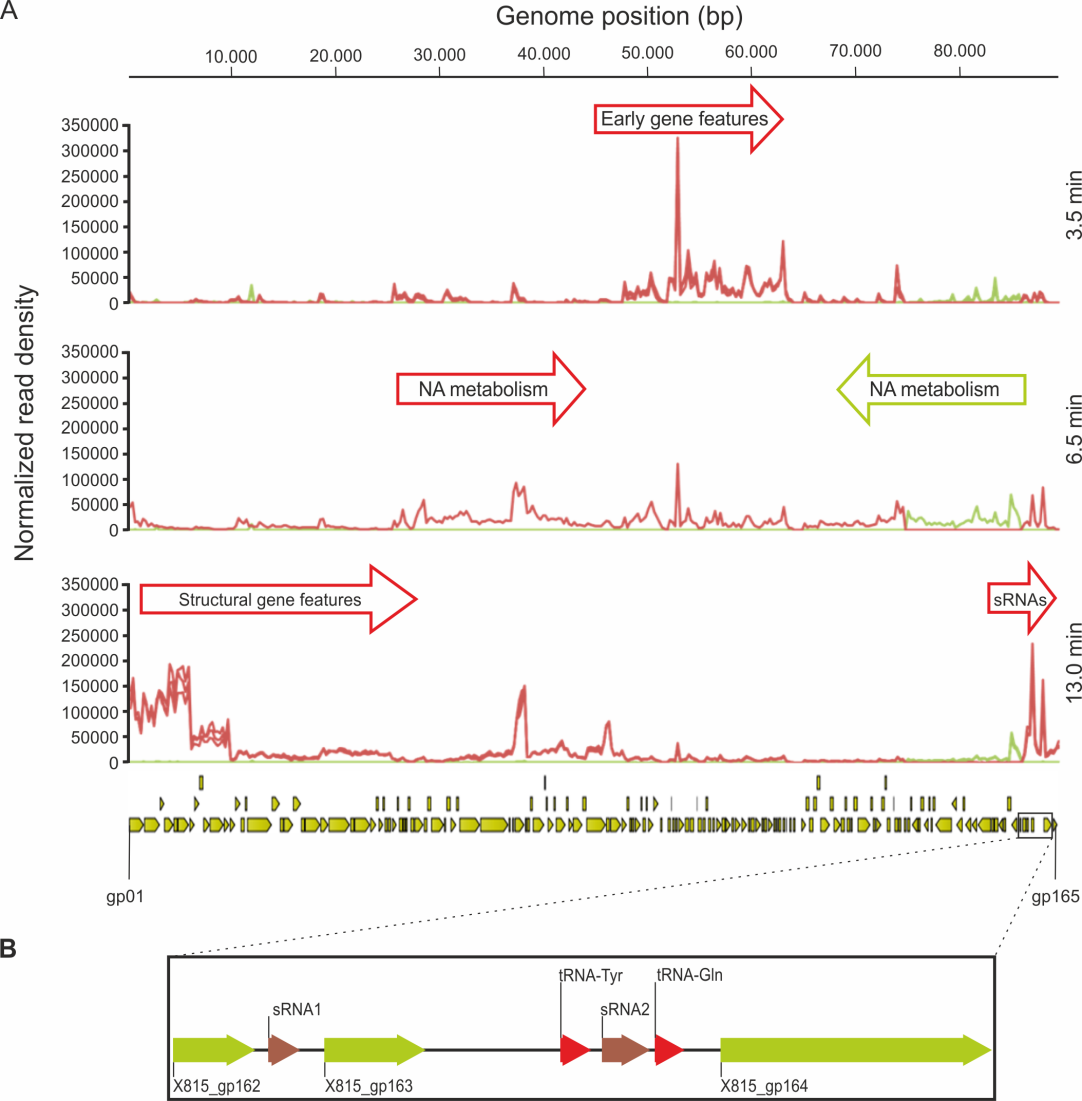
PAK_P3 transcription is temporally regulated. **(A)** Mapped reads were summarized into stranded count tables of Total Gene Reads that align to every 250bp of the PAK_P3 genome using the CLC Genomics Workbench. These read counts were then normalized against each other by the Total Count of reads that align to both phage and host genomes for each sample and then plotted. This allows us to show the relative abundance of phage transcripts over time in the context of the total transcript population. Red and green graphs represent reads mapping to the forward and reverse strands, respectively, for each replicate in each condition. The PAK_P3 genome is represented at the bottom of the panel with yellow arrows indicating defined coding sequences. **(B)** Enlargement of the PAK_P3 genome region encoding small RNAs. NA = nucleic acid

During the course of infection, early, middle and late transcripts of PAK_P3 genome were identified (Fig 7, S5 Table). The early transcribed region encompasses genes gp74 through gp112, all of which encode hypothetical proteins with low or no sequence similarity to gene products from other bacteriophages (so-called ‘ORFans’). Transcripts produced at middle time point focus on two regions that each contains gene features related to nucleic acid metabolism. As expected, the structural region appears to be mostly transcribed in late infection. Strikingly, five ORFs (*i*.*e*. gp34, gp37, gp38, gp45 and gp46), although located in the structural region, are overexpressed early compared to late time point. Finally, all predicted genes are transcribed, except for gp113, which corresponds to the predicted genome terminus. Intergenic transcription is observed throughout the genome, highlighting the great compaction of viral genomes where every single gene is expressed, in contrast to bacterial genomes. This property is further illustrated by the large amount of antisense transcripts detected, as reported below.

### PAK_P3 expresses antisense RNA elements targeting its structural region during the early stage of infection

Analysis of antisense transcripts of PAK_P3 revealed 20 putative asRNAs, 8 of which are small asRNA (mean length 176±30 bp) and 12 are longer than 300 bp (S6 Table). All but one are encoded within genes, suggesting they may act as *cis*-encoded antisense RNAs. These asRNA are predominantly (15 out of 20) located in the structural region of PAK_P3 genome and are significantly more strongly transcribed during early infection compared to late infection with fold changes ranging between 2 and 17 (S2 and S3 Figs, S5 Table). These data support the hypothesis that such antisense transcription is used to shut down expression of late structural genes during the early stage of infection.

### PAK_P3 displays strong expression of temporally regulated small, non-coding RNAs

Following the observation of abundant antisense transcripts, we looked for other unusual transcriptional profiles within PAK_P3 transcriptome and detected two abundant small (~100bp) transcripts during late infection. These two transcripts, hereafter referred as sRNA1 and sRNA2, were found in two neighboring intergenic regions: sRNA1 is encoded within a 200bp-intergenic region between two genes encoding hypothetical proteins, whereas sRNA2 is part of a larger intergenic region between two phage-encoded tRNAs (Fig 7). They are temporally regulated since they display a 91- and 12-fold change, respectively, in their ‘late versus early’ expression. Strikingly, these two small RNAs belong to the most strongly transcribed regions of the phage genome during late infection as they respectively represent the 18^th^ and 24^th^ most expressed gene features over 86 late genes (S5 Table).

We hypothesized that they could be *trans*-encoded small RNAs, acting by base-pairing on a target mRNA. As such, we looked for potential target regions in both phage and host genomes. The potential targets found on the host genome were not differentially expressed 13 min post infection, indicating that these two phage small RNAs would not act through mRNA degradation but rather have a role in translational silencing, if any.

Interestingly, a stretch of 11 nucleotides on sRNA2 was found to be repeated eight times on the host genome and systematically located within tRNAs, more particularly within the TψC-loop. We propose that it could be involved in translational repression by binding, and eventually blocking, bacterial ribosomes. As this 11bp-stretch is also conserved in closely related phages (*PAK_P1-like* genus), it may represent a starting point leading to the discovery of new phage non-coding RNAs.

On the phage genome, the only potential targets (11 consecutive nucleotides matching perfectly) are located in the early ORFan product gp78 for sRNA1 and in the late gene encoding the putative ribonucleotide-diphosphate reductase gp67 for sRNA2.

To date, only few phage-encoded small RNAs have been described in the literature and most of them derive from prophages [19,20]. The only examples of phage sRNAs encoded by a virulent phage, T4 band C and band D RNAs, were described in the 1970’s and their functions have remained unknown ever since [21].

## Discussion

Next generation transcriptomics, metabolomics, and classical microbiological techniques have here been integrated for the first time to describe virus/host interactions between the candidate therapeutic bacteriophage PAK_P3 and its host, *P*. *aeruginosa* strain PAK. By capturing early, middle and late infection time points, we delineated genomic regions of temporally distinct phage expression. This particularly highlights early gene features, which are typically involved in the shutdown of host metabolism. Like the approximately 50 so called ‘monkey-wrench’ proteins found in phage T4, small early proteins likely have functions reliant on protein-protein interactions to disrupt host systems and could potentially be exploited to aid in small molecule antibiotic design [22–24].

It is well established for model bacteriophages, including T7 and T4, that the temporal regulation of middle and late gene expression is typically the result of a tight regulation driven by phage early proteins through various mechanisms such as redirection of host RNA polymerase to phage middle and late promoters (like phage T4 proteins AsiA-MotA or phage-encoded sigma factor gp28 in SPO1) [16,25]. The early expression of antisense RNAs could represent an additional regulation mechanism preventing transcriptional leaks from strong promoters controlling expression of late structural genes. Consistent with this hypothesis, the temporal distribution and the location of the numerous PAK_P3 asRNAs correlate with the shut-off of structural gene expression observed 3.5 min post infection. Although *cis*-antisense RNAs appear to be a common form of regulation in bacterial genomes, they have not been extensively described in phage genomes. Beside the regulatory *oop* RNA reported over 40 years ago (reviewed in [26]), no other asRNAs were reported until recently [27] and exclusively in lambdoid phages. Moreover, such asRNAs have never been reported for virulent phages until 2014 [28]. Therefore, the high number asRNAs reported for PAK_P3 implies that antisense transcription may be a regulatory mechanism used by phages more frequently than previously thought.

From a phage-host interaction point of view, we found that existing host transcripts are rapidly overwhelmed with viral transcription. This may reflect a globally accelerated degradation of RNA in the cell in a way similar to phage T4. Indeed, it has been previously reported that T4 globally alters the stability of existing mRNAs, in addition to repressing the transcription of cytosine containing DNA [29,30]. This hypothesis is further supported by the drastic overexpression of one host operon (PAK_4493–4499) encoding RNA processing-related proteins. In particular, this operon encodes a RNA 3'-phosphate cyclase RtcA (PAK_4496) that has been described as being involved in the processing of RNA transcripts such as priming RNA strands for adenylylation to protect them from exonucleases or to mark them for further processing so they serve as substrates for downstream reactions performed by additional enzymes [31,32]. Therefore, we hypothesize that this operon may be uniquely upregulated by the phage in order to participate in the global degradation of RNAs during infection, which we observe in both the RNA-Seq and metabolomics data, by tagging transcripts for degradation by phage encoded enzymes. An alternative hypothesis to explain this dramatic up-regulation of PAK_4493–4499 relies on RtcB (PAK_4494), a predicted RNA ligase. Together, the RtcAB system has been shown to play a role in tRNA repair after stress-induced RNA damage (e.g. viral infection) in *E*. *coli* [33]. Also, it has been shown that phage T4 RNA 3'-phosphate cyclase (encoded by *pseT*) and RNA ligase (*rli*) are involved in overcoming resistance [34] by restrictive strains of *E*. *coli* producing phage induced tRNA anticodon nuclease (encoded by the *prr* locus) which causes abortive infection by preventing effective translation of phage genes [35]. Therefore, it is possible that PAK_P3 upregulates this operon to activate a host repair function to interfere with a yet uncharacterized host restriction system or a *prr*-like locus. However, deleting the RNA ligase *rtcB* gene (PAK_4494), appears to have no toxic effect on the host as well as no effect on the efficiency of plating (S1 Text).

**Fig 8:**
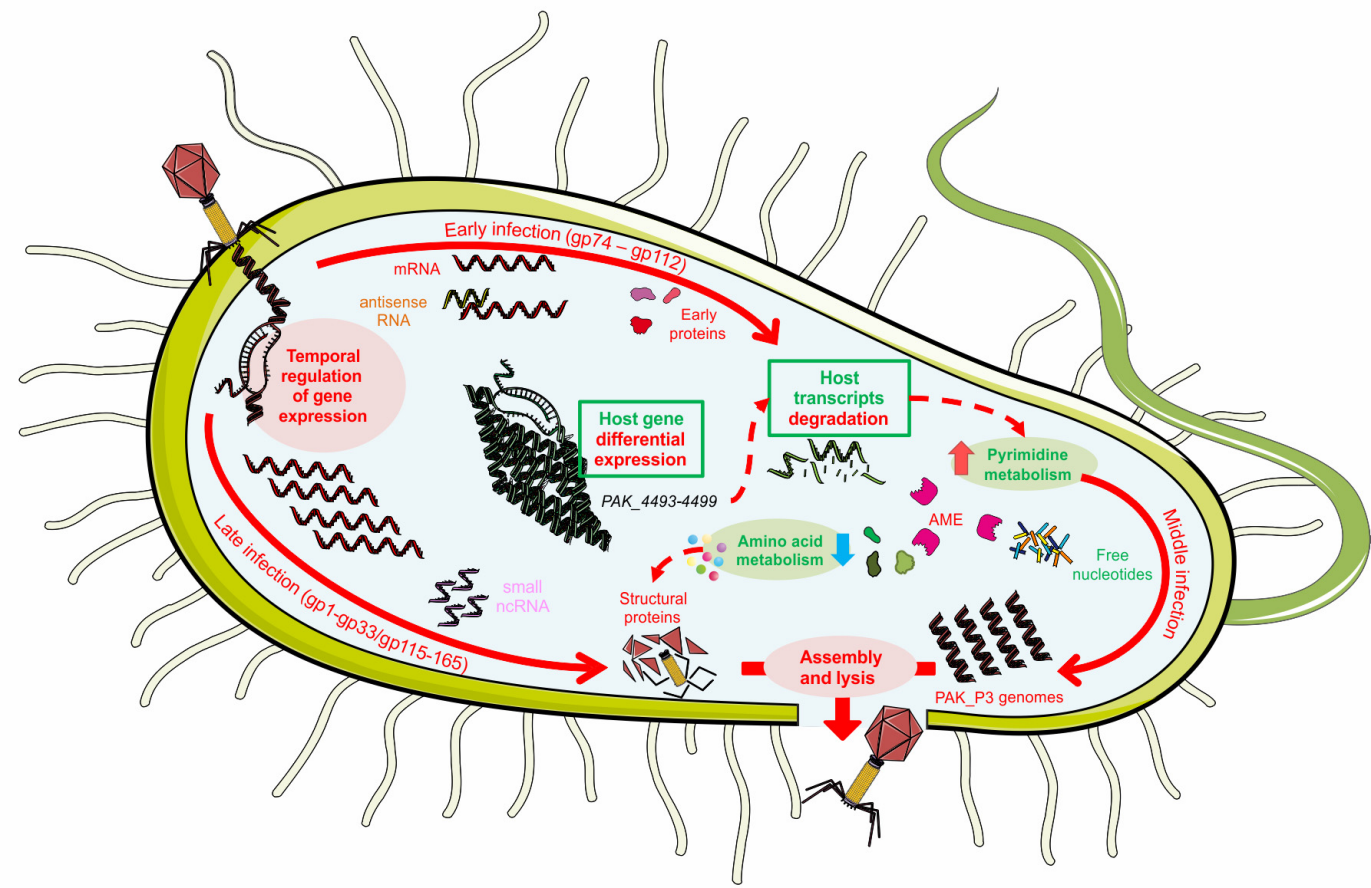
Molecular details of PAK_P3 infection cycle of *P*. *aeruginosa* strain PAK. Red and green colors correspond to phage and host elements respectively. A description of the figure is given in the text (see Discussion). Full arrows depict the three temporal stages of PAK_P3 infection cycle. Dashed arrows represent relations between metabolic pathways highlighted by our “-omics” analyses. AME = Auxiliary Metabolic Enzymes.

The observation of a phage induced host RNA degradation is further supported by our metabolomics data. Indeed, the increased pyrimidine metabolism confirms that nucleotide turnover is a central viral need to achieve a successful infection cycle. Overall, we showed that upon PAK_P3 infection, the host metabolism is not shutdown but redirected to generate the required building blocks for viral replication and this redirection is not the result of a phage induced differential host gene expression, aside from the RNA processing operon. An explanation for this metabolic turnover relies on phage-encoded AMGs and phage early proteins. We propose that phage early proteins would interfere with host metabolic processes through interactions with bacterial proteins. Once the host machinery is disrupted, phage metabolic enzymes would take over and catalyze the reactions yielding the specific metabolites required for viral replication (Fig 8). For instance, we hypothesize that the observed global degradation of host mRNA eventually produces an excess of free ribonucleotides that are likely converted into deoxynucleotides by ribonucleotidases. Interestingly, PAK_P3 encodes a putative ribonucleotide-diphosphate reductase (alpha and beta subunits, respectively gp67 and gp69) that could catalyze such a reaction. An alternative explanation for the observed increase of (deoxy)nucleotides-phosphates relies on the putative deoxyribonuclease (gp57) encoded by PAK_P3, which could be responsible for host genome degradation during middle and late infection stages, as observed for phage LUZ19 [36]. Supporting these hypotheses, these three phage-encoded AMGs are strongly expressed by PAK_P3 during late infection stage as they are respectively the 7^th^, 22^nd^ and 19^th^ most expressed genes over 86 late genes (S5 Table). It is noteworthy that a fourth predicted phage-encoded AMG, a CMP deaminase (gp155), is also involved in nucleotide metabolism and also expressed during late infection although less intensely than the other AMGs mentioned. Altogether, these predicted AMGs involved in nucleotide metabolism highlight the central need for nucleotides during PAK_P3 infection, in accordance with the short infection cycle span during which about 50 genomes of 88 kb have to be synthesized. Indeed, the advantage found in precisely manipulating nucleotide depolymerization and pathways to shut down host mechanisms, provide material for phage DNA synthesis, and prevent osmotic stress appears to be significant across phage clades. For example, *Pseudomonas* phage Lu11 contains ORFs predicted to be involved in nucleotide metabolism [37], *E*. *coli* phage T5 degrades host DNA before exporting it outside of the cell [38], and T4 even encodes for its own, nearly complete, parallel DNA precursor biosynthesis pathway [6].

Another example of phage-driven interference with host metabolic pathway is given by LPS biosynthesis pathway. The observed accumulation of cell wall precursors, which is not correlated with an altered expression of corresponding host genes, may be a direct consequence of peptidoglycan degradation and the subsequent release of its precursors triggered by the infection. Consistent with this hypothesis, PAK_P3 has a potential AMG (gp151) similar to a bacterial cell wall hydrolase, which could explain such cell-wall degradation.

Further investigations are now required to fully associate transcriptomics and metabolomics data to viral gene functions, a process which is currently hampered by the lack of versatile genetic tools to construct mutants of virulent bacteriophage. Such effort to deeply characterize one particular phage genus (i.e. *KPP10-like*) is also motivated by the great therapeutic potential of these phages as demonstrated in animal models and recently strengthened by the identification of such a phage in the ‘Intesti phage’ cocktail, a key commercial product of the Eliava Institute in Georgia [4,39–41]. Overall, the knowledge of phage biology provided by next-generation “-omics” approaches not only enlighten viral mechanisms of infection but can also open an array of biotechnological applications based on regulatory elements and proteins found in this new sub-family of phages.

## Materials and Methods

### Strains and growth conditions

*P*. *aeruginosa* strain PAK [42] was cultured in LB medium supplemented with 10mM MgCl_2_ at 37°C unless stated otherwise. For RNA-Seq experiments, cells were infected with bacteriophage PAK_P3 using a multiplicity of infection of 25 in order to ensure the synchronicity of the infection (95% of the bacterial population killed after 5 min phage-bacteria incubation).

### Adsorption assay and one-step growth experiment

Bacteriophage growth parameters were assessed as described previously [43]. Briefly, a culture of strain PAK was infected at low MOI (0.1) and incubated 5 min at 37°C with agitation allowing bacteriophage particles to adsorb. Following a 1000-fold dilution, two 100 μL samples were collected every 2 min and either kept on ice until titration, or mixed with CHCl_3_. For each time point we thus determined the free bacteriophage count (samples with CHCl_3_) as well as the number of free bacteriophages and infective centres (samples without CHCl_3_) to calculate eclipse and latency periods respectively.

Experimental data were fitted with a logistical function:

$$f(x)=\frac{a}{1+e^{-k(x-x_{c})}}$$ (1)

*a*: ordinate corresponding to the asymptote when x→+∞, represents the maximal pfu count.

*x*_*c*_: abscissa of the inflection point, represents the mean duration of the infectious cycle.

*k*: slope of the tangent line to the exponential part of the curve.

Eclipse and latency periods were determined as the *x* value corresponding to f(x)>0.05*a*

The burst size was determined as:

$$\frac{Phage\_titer_{\left(t=0,\text{-}CHCl_{3}\right)}\;\text{-}\;Phage\_titer_{\left(t=0,+CHCl_{3}\right)}}{Phage\_titer_{\left(t=30,\pm CHCl_{3}\right)}}$$ (2)

*Phage_titer*_*(t = 0*, *+CHCl3)*_ and *Phage_titer*_*(t = 0*, *-CHCl3)*_: values of initial phage titers (t = 0 min) measured in samples treated or not with chloroform, respectively. The numerator represents the number of intracellular phages.

*Phage_titer*_*(t = 30*, *±CHCl3)*:_ Mean of phage titers measured in samples treated and not treated with chloroform at t = 30 min.

Four independent adsorption assays were performed in the conditions described above with a lower MOI (10^−3^) and omitting the dilution step. Data could be approximated using an exponential function and adsorption time was defined as the time required to reach a threshold of 10% non-adsorbed bacteriophage particles.

### *P*. *aeruginosa* strain PAK genome sequencing

Genomic DNA was isolated from *P*. *aeruginosa* strain PAK and pyrosequencing was performed on a Roche 454 FLX system with Titanium chemistry at the University of Texas Genomic Sequencing and Analysis Facility. The draft assembly of ~6.3 Mbp consists of 9 scaffolds, 490 large contigs, and 616 total contigs and was annotated at the University of Maryland Institute for Genomic Sciences using the IGS Prokaryotic Annotation Pipeline[44]. Scaffolds deposited in GenBank can be accessed via Bioproject accession no. PRJNA232360. More details are available in S1 Text.

### Whole transcriptome sequencing

RNA-Seq analysis was performed on an exponentially growing culture that was synchronously infected with PAK_P3. Three independent biological replicates were harvested at 0min, 3.5min and 13min to represent, respectively, a phage negative control and early and late transcription while one additional sample was collected at 6.5 minutes to assess the presence of an identifiable middle phase of transcription.

The preparation of cDNA libraries was performed as described in Blasdel *et al*. (*in press*) [45]. Briefly, samples were collected at three time points, representing early, middle, and late infection, from a synchronously infected culture, with <5% of bacteria remaining uninfected after 3.5 minutes, and halted by rapid cooling in 1/10 volume of ‘stop solution’ (10% phenol, 90% ethanol). Cells were then lyzed in TRIzol, total RNA was purified through a standard organic extraction and ethanol precipitation, and remaining genomic DNA was removed using TURBO DNase. DNA removal was confirmed with PCR before rRNA was depleted using the Ribo-Zero rRNA Removal Kit (Gram-Negative Bacteria). This rRNA depleted total RNA was then processed into cDNA libraries using Illumina’s TruSeq Stranded Total RNA Sample Prep Kit according to manufacturer’s instructions and sequenced using an Illumina NextSeq 500 desktop sequencer on the High 75 cycle. More than 11 million 75bp reads mapping to non-ribosomal regions were obtained from each library with the exception of one early sample and one late sample providing 1,221,867 and 941,631 mapped reads respectively due to incomplete rRNA removal. After trimming, sequencing reads were aligned separately to both the phage and host genomes with the CLC Genomics workbench v7.5.1. These alignments were then summarized into count tables of Unique Gene Reads that map to phage or host gene features respectively.

RNA-Seq data have been deposited in NCBI-GEO with accession no. GSE76513.

RNA-Seq coverage visualization is available through the COV2HTML software at [https://mmonot.eu/COV2HTML/visualisation.php?str_id=-32] for a comparison of the host (0 min / 13 min) and [https://mmonot.eu/COV2HTML/visualisation.php?str_id=-34] for a comparison of the phage (3.5 min/ 13 min)

### Strain PAK reannotation and prediction of ncRNAs with RNA-Seq data

RNA-Seq data of uninfected strain PAK were visualized using COV2HTML [7]. Reads mapping forward and reverse strands were manually scanned over the whole genome. Both coding regions and intergenic regions displaying an unexpected transcription profile were examined using CLC Genomics Workbench 7.5.1 and Blastp (default parameters) to annotate putative new coding sequences or RNA central (http://rnacentral.org/sequence-search/), Rfam search (http://rfam.xfam.org/) and RNAfold web server (http://rna.tbi.univie.ac.at/cgi-bin/RNAfold.cgi) with default parameters to predict putative small RNAs and riboswitches.

### Statistical analysis

Each statistical comparison presented was performed using the DESeq2 [46] R/Bioconductor package to normalize samples to each other and then test for differential expression. Notably, we have chosen to normalize the population of reads that map to each genome independently of the other. In the context of a phage infected cell rapidly replacing host transcripts with phage transcripts, this has artificially enriched host reads and depleted phage reads progressively over the course of infection by normalizing away the biologically relevant shift in each organism’s proportion of the total reads in the cell. However, this has also allowed us to both show and test for differential expression of both phage and host gene features independently of the more global swing towards phage transcription.

### High coverage metabolomics analysis

*P*. *aeruginosa* strain PAK cells, grown in minimal medium (30 mM Na_2_HPO_4_, 14 mM KH_2_PO_4_, 20 mM (NH_4_)_2_SO_4_, 20 mM glucose, 1 mM MgSO_4_, 4 μM FeSO_4_), were infected with PAK_P3 at OD_600_ = 0.3 (approx. 1.25.10^8^ CFU). At 0, 5, 10, 15, 20, and 25 minutes post infection, cells were collected by fast filtration [47]. The biomass quantity was adjusted to match the biomass of a 1 mL culture at OD_600_ = 1.0 (approx. 4.10^8^ CFU) by following the OD_600_ and adjustment of the sampling volume. Four biological replicates were sampled and two technical repeats were made of each independent biological sample. The metabolic content was extracted as described by De Smet *et al* [3]. The samples were profiled using only negative mode flow injection-time-of-flight mass spectrometry and detected ions were annotated as previously reported [17]. Metabolite annotation and statistical analysis was performed using Matlab R2013b (Mathworks, Natick, MA, United States) according to the ion annotation protocol described by Fuhrer *et al*. [17]. With this method, 6006 ions were detected and 918 of them could be assigned to known *P*. *aeruginosa* metabolites. After removal of ion adducts, 377 ions were retained that were annotated as 518 metabolites (including mass isomers).

Differential analysis was performed for each time point versus time point zero using a t-test for two samples with unequal variances (Welch test). For metabolic pathway enrichment, lists of significantly changing metabolites for each time point were created based on the thresholds of │Log_2_(fold change)│ ≥ 0.5 and adjusted p-value < 0.1. In each list, metabolites were sorted by the adjusted p-value, and the pathway enrichment procedure was performed for each subset of size 1 to the size of the significant list using Fischer test as described in [48] and the smallest p-value for each pathway was reported. For differential analysis and pathway enrichment, p-values were adjusted for multiple hypotheses testing with the Benjamini-Hochberg procedure.

## Supporting Information

S1 Text Supplemental methods. (DOCX)

S1 Table *P*. *aeruginosa* strain PAK reannotation based on transcriptomic data of the uninfected sample. Newly annotated features (small RNAs, riboswitches, CDS and antisense RNAs) are listed. Their location on PAK genome, their sequence and comments on their annotation are indicated.
(XLSX)

S2 Table *P*. *aeruginosa* strain PAK differential gene expression statistical analysis. This table lists all PAK annotated features as well as their corresponding mean counts normalized with the DESeq2 normalization method while excluding phage counts, the calculated fold change and the results of a DESeq2 test for differential expression comparing the non-infected (0 min) and infected (13 min) conditions.
(XLSX)

S3 Table PAK_P3 infection causes a dramatic increase in free deoxynucleotides. This table shows the Log_2_(fold change) for all measured nucleotides over the course of PAK_P3 infection. In grey are those not significantly influenced, the color scale indicates the level of increase (green) or decrease (red) of the metabolite at the specific time point. Metabolite levels displaying more than 2-fold increase are highlighted in bold.
(XLSX)

S4 Table PAK_P3 infection influences amino sugar metabolism. This table shows the Log_2_(fold change) for all indicated metabolites over the course of PAK_P3 infection. In grey are those not significantly influenced, the green color scale indicates the level of increase of the metabolite at the specific time point.
(XLSX)

S5 Table PAK_P3 differential gene expression statistical analysis. This table lists all PAK_P3 annotated features and their corresponding total gene reads as well as those phage counts for early (3.5 min) and late (13 min) infection normalized against each other, excluding host reads. Values for each independent biological replicate (R1, R2, R3) are indicated and the results of our statistical analysis are provided.
(XLSX)

S6 Table PAK_P3 antisense transcripts and small RNAs. Newly annotated RNA features (small RNAs and antisense RNAs) are listed. Their location on PAK_P3 genome, their sequence and comments on their annotation are indicated.
(XLSX)

S1 Fig Strain PAK displays high number of non-coding RNAs. A representative example of detection of **(A)** intergenic small RNA (sRNA); **(B)** riboswitch (RSw) and **(C)** *cis*-antisense RNA (asRNA). The mapped reads were formatted into graph files for visualization in a strand-specific manner (black and pink represent reads mapping the forward and the reverse strands, respectively) using COV2HTML. The annotated non-coding RNA genes are indicated as grey arrows and open-reading frames annotated in strain PAK are shown as blue arrows.
(TIF)

S2 Fig Temporal regulation of bacteriophage PAK_P3 gene expression is revealed by RNA-Seq analysis. Differential expression analysis comparing the expression of phage gene features between early (t = 3.5 min) and late infection (t = 13 min) while excluding host reads from the normalization. Blue = Early gene features, Orange = Late gene features, Red = Gene features not significantly differentially expressed. The previously annotated gp113 is also shown with negligible expression throughout infection and has been deleted from the annotation.
(TIF)

S3 Fig Bacteriophage PAK_P3 expresses many antisense transcripts. Mapped reads were formatted into graph files for visualization in a strand-specific manner (black and pink represent reads mapping the forward and the reverse strands, respectively) using COV2HTML. The annotated *cis*-antisense RNAs genes are indicated as grey arrows and open-reading frames annotated in PAK_P3 are shown as blue arrows. Data obtained 3.5 min and 13 min following PAK_P3 infection are presented in upper and lower part, respectively.
(TIF)
