# Supplementary material for: Next-Generation “-omics” Approaches Reveal a Massive Alteration of Host RNA Metabolism during Bacteriophage Infection of Pseudomonas aeruginosa
Source: PLoS Genet. 2016 Jul 5;12(7):e1006134. doi: 10.1371/journal.pgen.1006134 (PMC4933390; doi:10.1371/journal.pgen.1006134)
Supplement: S1 Text — (DOCX) [file pgen.1006134.s001.docx]

***P. aeruginosa* strain PAK genome sequencing**

Genomic DNA was isolated from *P. aeruginosa* strain PAK using a DNeasy Blood and Tissue Kit (Qiagen). DNA was fragmented to 3 kb fragments for mate-paired library preparation by hydroshearing, followed by AMPure bead purification (Beckman Coulter). Fragment size was verified by Bioanalyzer (Agilent). DNA fragments were end polished with T4 DNA polymerase and T4 PNK (NEB) and purified using a QIAquick spin column (Qiagen). Roche circularization adapters were ligated to genomic DNA fragments with NEB quick ligase and purified with QIAquick spin columns. AMPure beads were used to size select DNA. A fill-in reaction was carried out with Bst polymerase and reactions products were purified with QIAquick spin columns. DNA fragments were circularized using Cre recombinase, while remaining linear DNA fragments were degraded by treatment with Plasmid-safe DNase and exonuclease I, followed by clean-up on QIAquick spin columns. Circularized DNA was fragmented by nebulization in nebulization buffer at 32, 40, and 42 psi for 2 m each to achieve the desired DNA fragment sizes for sequencing. After each nebulization step DNA fragments were purified with MinElute columns (Qiagen) and sizes were determined by Bioanalyzer. Fragmented DNA was end-polished as described above and purified with QIAquick spin columns. Purified fragments were immobilized on Dynabeads M-270 streptavidin beads (Life Technologies), and library adapters were ligated to immobilized fragments with NEB quick ligase. Adapter-ligated library fragments were washed and fill-in reactions were performed on immobilized library fragments, as described above. The immobilized library was washed and PCR amplified with library amplification primers (Roche) using GC-rich enzyme mix (Roche). Final library size selection was performed by AMPure bead purification. The size-selected library was bound to Dynabeads M-270 streptavidin beads rotating for 15 m at 25°C. Finally, ssDNA library fragments were eluted from beads with 0.125 N sodium hydroxide and purified with MinElute spin columns. Pyrosequencing was performed on a Roche 454 FLX system with Titanium chemistry at the University of Texas Genomic Sequencing and Analysis Facility and generated 634,299 raw sequencing reads for 138,694,143 bp. Using the mate-paired information, reads were assembled into contigs and scaffolds with the Newbler assembler software version 2.3. The draft assembly of ~6.3 Mbp consists of 9 scaffolds, 490 large contigs, and 616 total contigs. The n50 scaffold size was 1,405,330 bp and the mean scaffold size was 713,656 bp. The n50 contig size was 27,011 bp and the mean contig size was 12,860 bp. The draft genome was annotated at the University of Maryland Institute for Genomic Sciences using the IGS Prokaryotic Annotation Pipeline [1].

The draft genome sequence of *P. aeruginosa* strain PAK has been deposited in GenBank with accession no. KK037225.1.

**Construction of Pseudomonas PAK *rtcB* deletion mutant**

In-frame, scarless deletion of PAK_4494 (*rtcB*) gene was performed with two-step allelic exchange as described by Hmelo *et al*. (2015) [2]. Briefly, upstream and downstream regions (~500bp length using Down and Up primers indicated below) of *rtcB* were PCR amplified and assembled by splicing by-overlap extension-PCR. This deletion allele was cloned into the allelic exchange vector pDONRPEX18Gm subsequently transformed in a conjugative *E. coli* strain (e.g. S17λpir) and ultimately transferred to *P. aeruginosa* PAK by biparental mating. The deletion mutants were finally selected on no-salt LB agar containing 15% sucrose and checked by Sanger sequencing of the region of interest using Seq_F and Seq_R primers indicated below.

| Primer name | Sequence (5’- 3’) |
| --- | --- |
| Down_F | AAGCCGATCAAGCTCTGGACCCTGCGTCAGGTGGTGTGCGTG |
| Down_R | GGGGACCACTTTGTACAAGAAAGCTGGGTAGTACGGAGTGAAAGGCGCTGC |
| Up_F | GGGGACAAGTTTGTACAAAAAAGCAGGCTCACTCGACGAACTGCTGGAGAAC |
| Up_R | GGTCCAGAGCTTGATCGGCTT |
| Seq_F | GAGTATCTGTACCGCGAGC |
| Seq_R | GATGGCGTTCTTCCCTGTTC |

**Table: Primers used to construct *rtcB* deletion mutant**

PAK_P3 efficiency of plating and lysis kinetics on PAK*∆rtcB* strain were assessed as described in ref [3]. No difference with the PAK wild type strain was observed.

**References**

1. Galens K, Orvis J, Daugherty S, Creasy HH, Angiuoli S, et al. (2011) The IGS Standard Operating Procedure for Automated Prokaryotic Annotation. Stand Genomic Sci 4: 244-251.

2. Hmelo LR, Borlee BR, Almblad H, Love ME, Randall TE, et al. (2015) Precision-engineering the *Pseudomonas aeruginosa* genome with two-step allelic exchange. Nat Protoc 10: 1820-1841.

3. Henry M, Lavigne R, Debarbieux L (2013) Predicting In Vivo Efficacy of Therapeutic Bacteriophages Used To Treat Pulmonary Infections. Antimicrobial Agents and Chemotherapy 57: 5961-5968.
