# Supplementary figures and images for: Next-Generation “-omics” Approaches Reveal a Massive Alteration of Host RNA Metabolism during Bacteriophage Infection of Pseudomonas aeruginosa
Source: PLoS Genet. 2016 Jul 5;12(7):e1006134. doi: 10.1371/journal.pgen.1006134 (PMC4933390; doi:10.1371/journal.pgen.1006134)

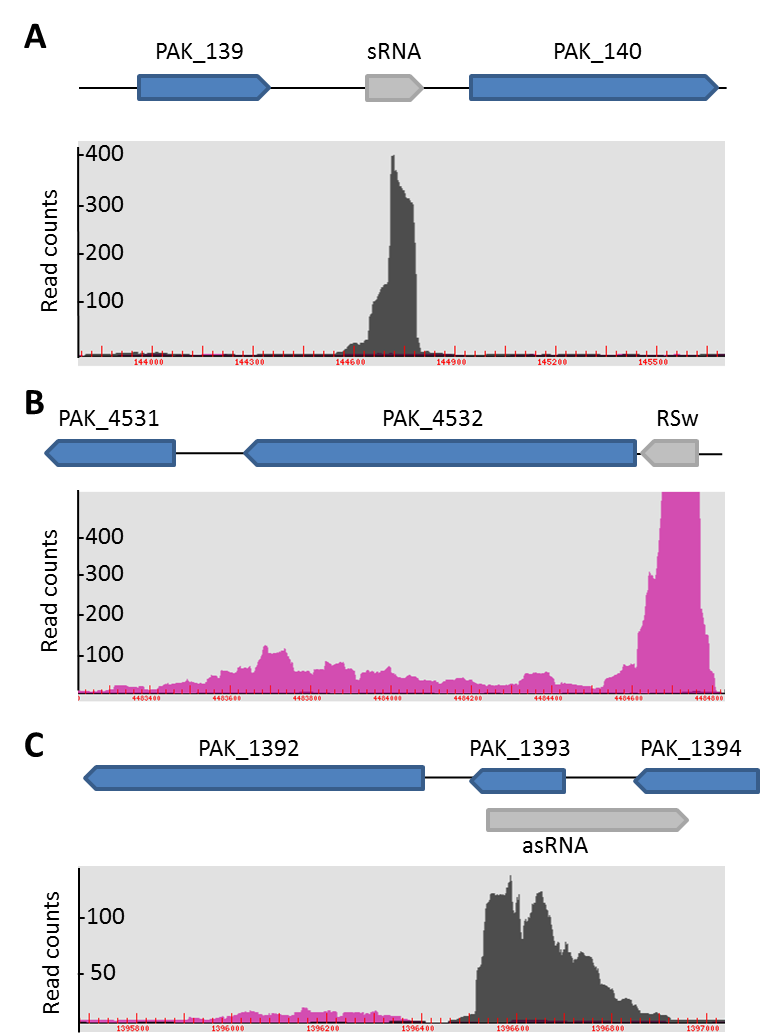

Supplement: S1 Fig — A representative example of detection of (A) intergenic small RNA (sRNA); (B) riboswitch (RSw) and (C) cis-antisense RNA (asRNA). The mapped reads were formatted into graph files for visualization in a strand-specific manner (black and pink represent reads mapping the forward and the reverse strands, respectively) using COV2HTML. The annotated non-coding RNA genes are indicated as grey arrows and open-reading frames annotated in strain PAK are shown as blue arrows. (TIF) [file pgen.1006134.s008.tif]

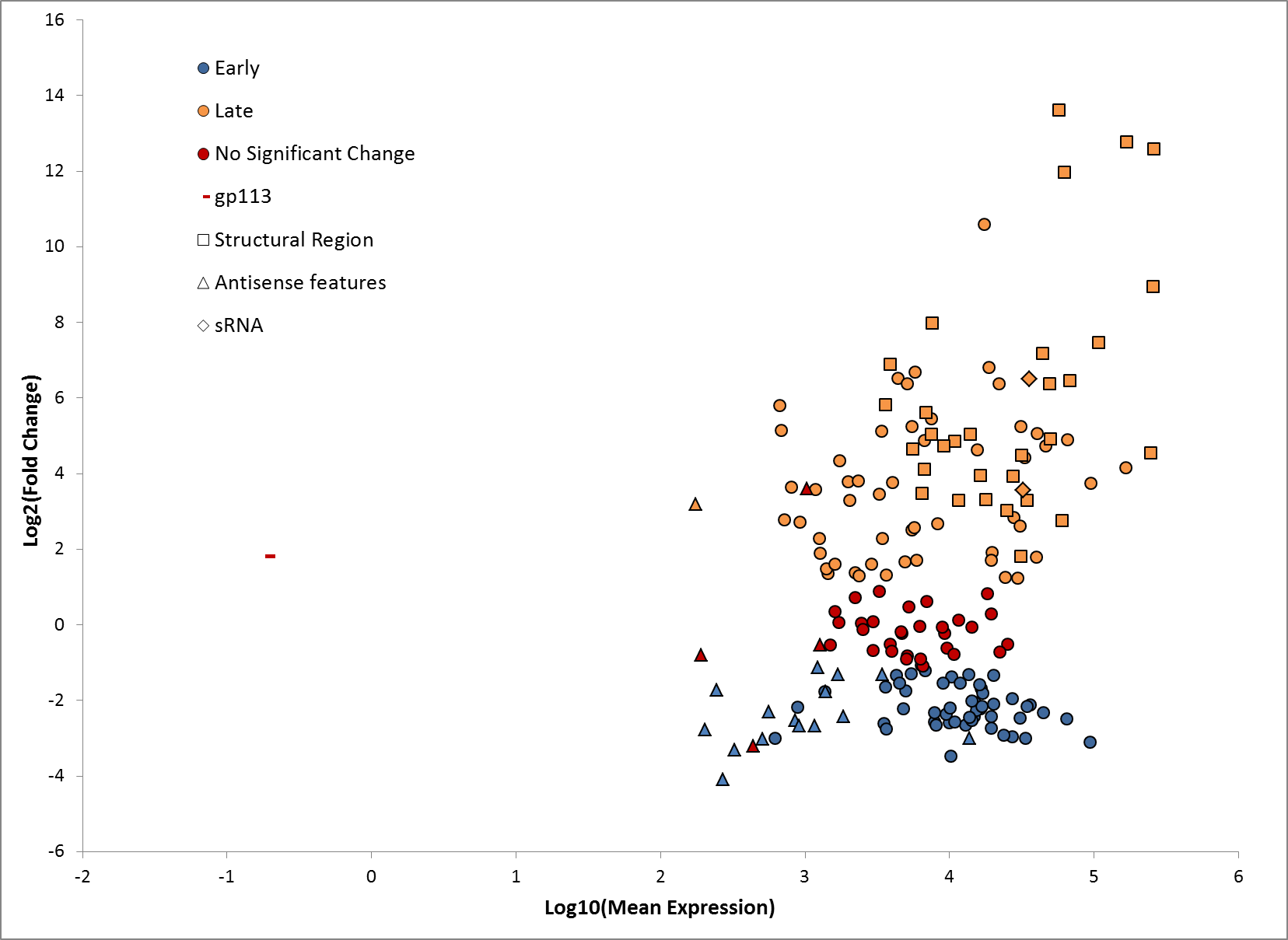

Supplement: S2 Fig — Differential expression analysis comparing the expression of phage gene features between early (t = 3.5 min) and late infection (t = 13 min) while excluding host reads from the normalization. Blue = Early gene features, Orange = Late gene features, Red = Gene features not significantly differentially expressed. The previously annotated gp113 is also shown with negligible expression throughout infection and has been deleted from the annotation. (TIF) [file pgen.1006134.s009.tif]

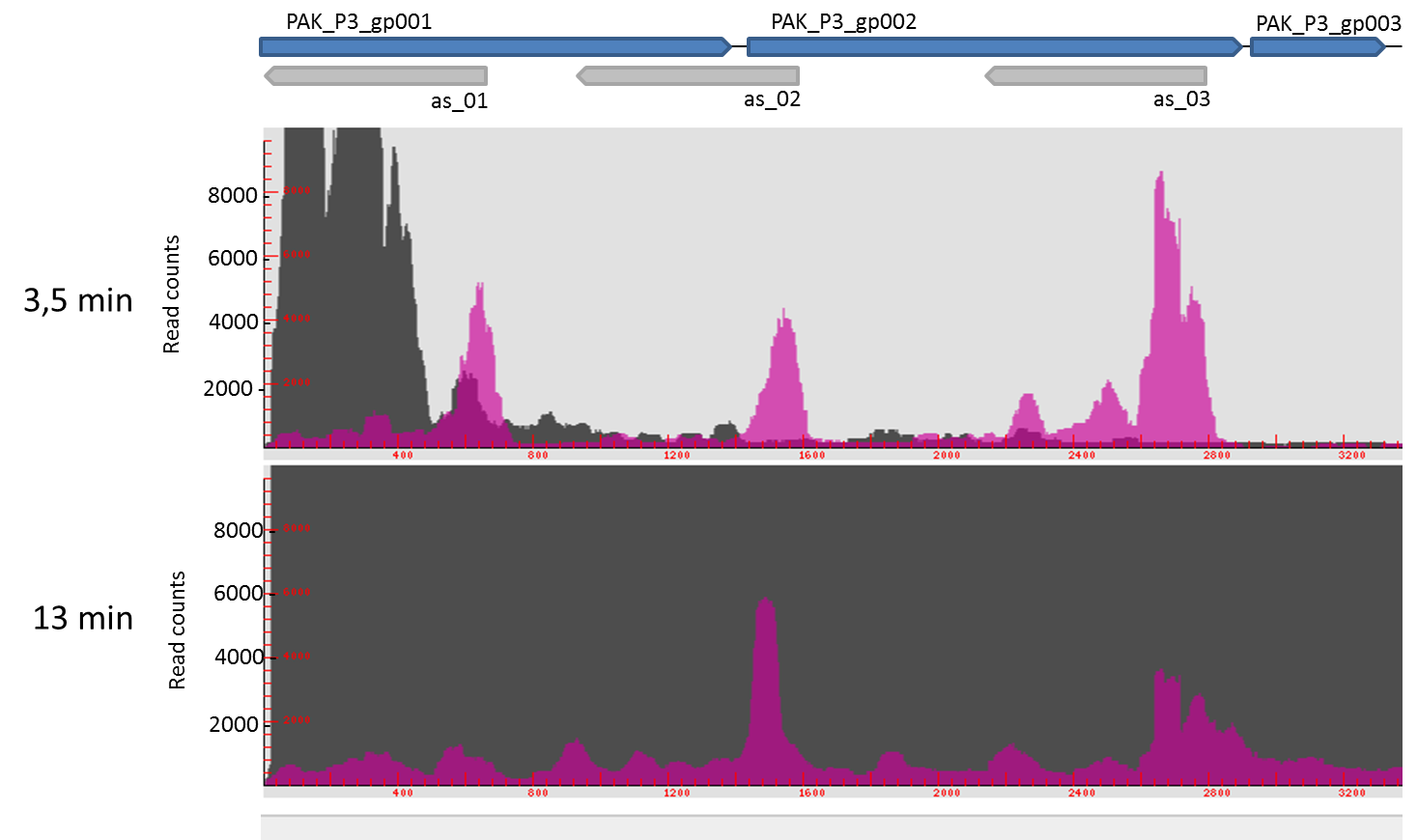

Supplement: S3 Fig — Mapped reads were formatted into graph files for visualization in a strand-specific manner (black and pink represent reads mapping the forward and the reverse strands, respectively) using COV2HTML. The annotated cis-antisense RNAs genes are indicated as grey arrows and open-reading frames annotated in PAK_P3 are shown as blue arrows. Data obtained 3.5 min and 13 min following PAK_P3 infection are presented in upper and lower part, respectively. (TIF) [file pgen.1006134.s010.tif]
